# Artificial intelligence uncertainty quantification in radiotherapy applications — A scoping review

**DOI:** 10.1016/j.radonc.2024.110542

**Published:** 2024-09-17

**Authors:** Kareem A. Wahid, Zaphanlene Y. Kaffey, David P. Farris, Laia Humbert-Vidan, Amy C. Moreno, Mathis Rasmussen, Jintao Ren, Mohamed A. Naser, Tucker J. Netherton, Stine Korreman, Guha Balakrishnan, Clifton D. Fuller, David Fuentes, Michael J. Dohopolski

**Affiliations:** a Department of Imaging Physics, The University of Texas MD Anderson Cancer Center, Houston, TX, USA; b Department of Radiation Oncology, The University of Texas MD Anderson Cancer Center, Houston, TX, USA; c Research Medical Library, The University of Texas MD Anderson Cancer Center, Houston, TX, USA; d Department of Oncology, Aarhus University Hospital, Denmark; e Department of Radiation Physics, University of Texas MD Anderson Cancer Center, Houston, TX, USA; f Rice University, Houston, TX, USA; g Department of Radiation Oncology, The University of Texas Southwestern Medical Center, Dallas, TX, USA

## Abstract

**Background/purpose:**

The use of artificial intelligence (AI) in radiotherapy (RT) is expanding rapidly. However, there exists a notable lack of clinician trust in AI models, underscoring the need for effective uncertainty quantification (UQ) methods. The purpose of this study was to scope existing literature related to UQ in RT, identify areas of improvement, and determine future directions.

**Methods:**

We followed the PRISMA-ScR scoping review reporting guidelines. We utilized the population (human cancer patients), concept (utilization of AI UQ), context (radiotherapy applications) framework to structure our search and screening process. We conducted a systematic search spanning seven databases, supplemented by manual curation, up to January 2024. Our search yielded a total of 8980 articles for initial review. Manuscript screening and data extraction was performed in Covidence. Data extraction categories included general study characteristics, RT characteristics, AI characteristics, and UQ characteristics.

**Results:**

We identified 56 articles published from 2015 to 2024. 10 domains of RT applications were represented; most studies evaluated auto-contouring (50 %), followed by image-synthesis (13 %), and multiple applications simultaneously (11 %). 12 disease sites were represented, with head and neck cancer being the most common disease site independent of application space (32 %). Imaging data was used in 91 % of studies, while only 13 % incorporated RT dose information. Most studies focused on failure detection as the main application of UQ (60 %), with Monte Carlo dropout being the most commonly implemented UQ method (32 %) followed by ensembling (16 %). 55 % of studies did not share code or datasets.

**Conclusion:**

Our review revealed a lack of diversity in UQ for RT applications beyond auto-contouring. Moreover, we identified a clear need to study additional UQ methods, such as conformal prediction. Our results may incentivize the development of guidelines for reporting and implementation of UQ in RT.

## Introduction

Artificial intelligence (AI) in healthcare has become increasingly important due to its potential to enhance diagnosis, treatment, and prognostic prediction [1]. A significant obstacle to the clinical implementation of AI that is receiving growing attention is a relative absence of model uncertainty quantification (UQ) [2], i.e., measures of predictive confidence. The ability of an AI model to characterize and communicate its uncertainty, in other words, learning when to say “I don’t know” [3], could enhance clinician trust and facilitate the integration of AI into clinical practice [2,4,5].

Radiotherapy (RT) is a fundamental pillar of cancer treatment used in approximately 50 % of all malignancies [6]. Due to the highly quantitative and structured nature of the RT clinical workflow, AI-based methodologies — namely, machine learning (ML) and deep learning (DL) — have been increasingly investigated to automate and improve a variety of tasks [7]. Advances in DL algorithms trained on increasingly larger, diverse datasets have allowed for impressive performance in a variety of RT-related applications such as image synthesis [8], registration [9], contouring [10], dose prediction [11], and outcome prediction [12–14]. However, despite the impressive performance of these models in research studies, to date there are relatively few standard AI-based tools that are routinely used in RT workflows. This hesitation could be partially attributed to insufficient clinician trust [15,16]. Enhanced UQ could bridge this trust gap, fostering greater confidence in AI applications within the RT field.

Conventionally there are two types of uncertainty: aleatoric and epistemic [17]. Aleatoric uncertainty arises from the noise inherent in the data. An example is the inherent variation in contour “ground truth” among radiation oncologists, each can be “right” but likely slightly different [18]. Epistemic uncertainty stems from incomplete information. For instance, a head and neck tumor contouring model may have limited exposure to certain rare malignancies (e.g., salivary gland cancer) and may generate poor contours with high epistemic uncertainty as these cases were underrepresented in model development. Models trained outside of medicine are often trained on datasets with > 1 million samples [19]. Medical datasets, especially in RT, are considerably smaller [20], often ranging from hundreds to thousands of patient samples. Thus, epistemic uncertainty estimation would be particularly important for RT model development. Together, aleatoric and epistemic uncertainty account for the total predictive uncertainty [21], though recent literature has begun to challenge this convention [22]. Illustrative figures related to aleatoric and epistemic uncertainty concepts are shown in Appendix A Figure A1.

Within the UQ literature, there exists several methods for providing estimates of uncertainty. Contemporary methods for estimating uncertainty in ML often adopt a Bayesian perspective, treating model predictions as probability distributions rather than single point values. For instance, when predicting if a patient will develop xerostomia after radiation therapy, the model might output an 80 % probability instead of simply stating “yes” or “no”. These probabilistic measures could enable safer model deployment in various clinical applications [21]. For example, UQ could be used in auto-segmentation for failure detection, flagging cases with a low probability of an accurate segmentation (i.e., high uncertainty) for additional clinical review. UQ methods such as Monte Carlo dropout and ensembles, which are suggested to be grounded in Bayesian principles [23,24], have surged in popularity in recent years [25]. However, emerging techniques, such as conformal prediction [26], are increasingly drawing on more traditional statistical methodologies.

Finally, worthy of note is that UQ has historically been closely linked to calibration, which measures the agreement between predicted probabilities and observed frequencies. Large-scale ML models — particularly DL models with numerous parameters — often show poor calibration, with output probabilities being higher than observed probabilities, subsequently leading to overconfident predictions [27]. UQ methods can help quantify and mitigate poor calibration; for example Monte Carlo dropout and ensembles often inherently improve confidence calibration [28,29]. For readers interested in more technical reviews on UQ concepts generally and specific to RT, we refer to comprehensive narrative works by Hullermeier & Waegeman et al. [17] and van den Berg & Meliadò [30], respectively.

While previous systematic and scoping reviews have covered the topics of UQ in healthcare generally [25,31] and in relation to medical imaging [32–34], these studies lacked any explicit focus on RT-related applications. Therefore, we conducted this scoping review to synthesize current trends for UQ in RT and provide an outlook for the future of this important research area for clinicians and researchers. An overview of our study is illustrated in Appendix A Figure A2.

## Materials and methods

This scoping review was conducted in line with the reporting guidelines of Preferred Reporting Items for Systematic Reviews and Meta-Analyses extension for Scoping Reviews (PRISMA-ScR) [35]. The pre-registration for this scoping review was performed using the Open Science Foundation Generalized Systematic Review Registration template and can be found online (https://doi.org/10.17605/OSF.IO/E3DQG). We utilized Covidence [36] — a standardized web-based literature review collaboration software platform — to perform all initial study screening and data extraction.

### Eligibility criteria

This scoping review was conducted to summarize the state of literature that implemented AI UQ for RT. We utilized the population, concept, context (PCC) framework to develop a focus question as recommended by the Joanna Briggs Institute Scoping Review Methodology Group [37]. Population was defined as human patients undergoing RT for cancer treatment, concept was defined as utilization of AI and UQ, and context was defined as RT applications (e.g., image acquisition and synthesis, tumor and organ at risk contouring, dose prediction, outcome prediction, etc.). Additional details on the PCC eligibility criteria and its integration into the search strategy are discussed in Appendix B.

### Search strategy

A medical research librarian (D.P.F.) searched MEDLINE (Ovid), Embase (Ovid), PubMed (NLM), Cochrane Library (Wiley), and Web of Science Core Collection (Clarivate) from inception to November 17, 2023 with a supplementary search of Web of Science Preprint Citation Index (Clarivate) and Google Scholar (Alphabet Inc.) from inception to December 12, 2023. A total of 8974 results were retrieved from the five databases including an original set of 9 key articles supplied by the research team (MEDLINE=1084; Embase = 1708; PubMed = 1154; Cochrane = 42; Web of Science Core Collection = 4358; Web of Science Preprint Citation Index = 428; Google Scholar = 200). The full search strategy details and inputs for each database is available in Appendix C. Notably, we incorporated 6 additional manuscripts that were not captured in the initial eligibility screening post-hoc via manual citation searching up to January 19, 2024; these manuscripts were principally added because they were formally indexed after the initial search date and were deemed relevant to ensure a more up-to-date review. Search results were uploaded to Covidence; after deduplication, 6017 unique results were identified for eligibility screening. The full PRISMA-ScR flow diagram is shown in Appendix A Figure A3.

### Study selection

Initial screening to ensure studies broadly fit within our defined PCC framework was performed in Covidence by 2 independent reviewers (K. A.W., Z.Y.K.) based on titles and abstracts; a third reviewer (M.J.D.) was consulted when disagreements occurred. A second full text review of these articles was performed to ensure all inclusion criteria were fully satisfied. Additional details on study selection are presented in Appendix B. Only full English-language preprints, conference proceedings, and standard peer-reviewed publications were included for this study; conference abstracts, editorials, review papers, and graduate theses were excluded. Conference proceedings and preprints were deemed appropriate for inclusion due to their ubiquitous nature in computational fields [38]. Preclinical and animal studies were not included in this review. 56 articles were ultimately selected for final inclusion (Appendix A Figure A3).

### Data extraction

Two reviewers extracted data from the final manuscripts (K.A.W., Z. Y.K.); a third reviewer (M.J.D.) was consulted when disagreements occurred. Additional details on data extraction are presented in Appendix B. Data were initially extracted using a template generated in Covidence, focusing on four categories: general study characteristics, RT characteristics, AI characteristics, and UQ characteristics. General study characteristics included manuscript type, publication year, geographic location of the study authors, and code/data availability. RT characteristics included intended RT application space (e.g., contouring, dose planning, etc.), specific data types used (e.g., CT, MRI, etc.), and patient cancer type. AI characteristics included algorithmic approach, training/validation/testing sample sizes, and properties of the validation/testing (e.g., separate set, cross-validation, etc.). AI characteristics were adapted from existing related guidelines including TRIPOD [39] and CLAIM [40]. UQ attributes included application category, method type, evaluation metrics, self-described uncertainty type (i.e., aleatoric vs. epistemic), and use of quantitative or qualitative evaluation methods. UQ application categories and definitions were adapted from Kahl et al. [21] and Lambert et al. [34]. Briefly, UQ application categories included active learning (improving training with uncertainty estimates), ambiguity modeling (comparing model and ground truth uncertainty), calibration (aligning model and true probabilities), failure detection (flagging incorrect predictions), and out-of-distribution detection (identifying distribution shifts from training data). Additional specific considerations for each category in the data extraction process are described in detail in Appendix B. To assess method suitability in contouring studies, we conducted an additional data extraction based on failure detection recommendations from the ValUES framework — a systematic investigation of segmentation UQ — by Kahl et al. [21] (Appendix D).

### Analysis

The final extracted data were analyzed using Python v. 3.10. Descriptive statistics and visual plots were generated using the pandas, seaborn, matplotlib, numpy, geopandas, and squarify Python libraries. We also compared the overlap of extracted publications in our study and publications extracted in previous systematic and scoping reviews in similar topic domains (i.e., UQ in medical applications). To accomplish this, we compiled a comprehensive list of all publications referenced in these studies during the data extraction process along with their respective titles and digital object identifiers (DOIs). Initially, we attempted to automatically match DOIs from studies in our scoping review with those in the existing literature. If no DOI match was found, we proceeded to automatically compare titles using the difflib Python library, setting a sequence match ratio threshold of at least 0.75. All identified matches were then subsequently manually verified.

### Data and code availability

A CSV file containing the final studies and corresponding extracted data for this scoping review are made publicly available through Figshare (https://doi.org/10.6084/m9.figshare.25535017.v1). All Python code used in the analysis can be found on Github (URL: https://github.com/kwahid/RT_UQ_scoping_review/tree/main).

## Results

Table 1 presents an overview of the extracted data from the final 56 manuscripts included in this review.

**Fig. 1.**
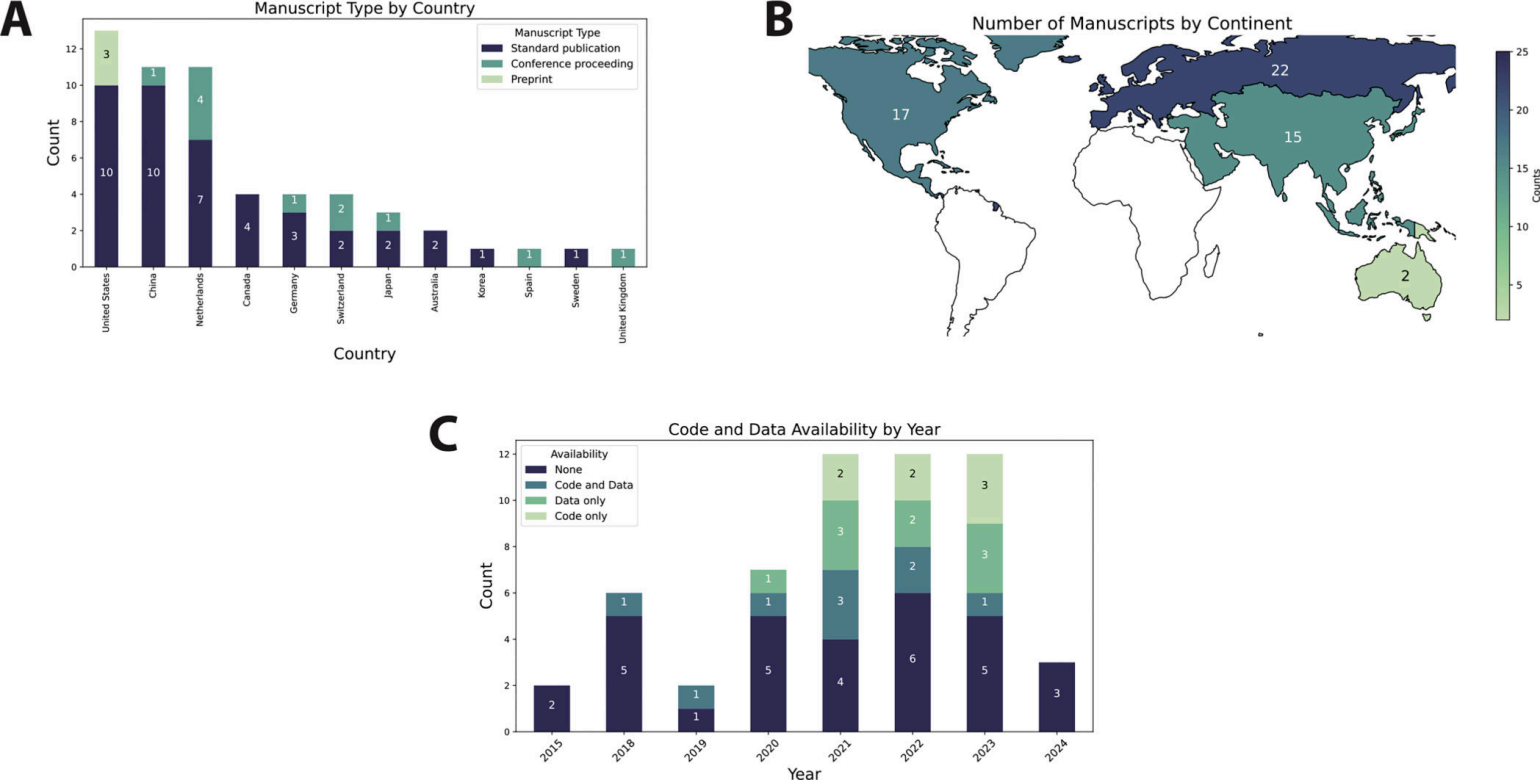
General study characteristics. (**A**) Stacked barplot showing total number of publications per country by publication type. (**B**) Heatmap of the number of studies by continent where green indicates a low number of publications and blue indicates a high number of publications; continents where no studies were extracted from are represented in white. (**C**) Stacked barplot showing code and data availability over time. Each item in the barplots corresponds to one study. (For interpretation of the references to colour in this figure legend, the reader is referred to the web version of this article.)

Twelve countries of origin were represented, with the majority of studies emanating from the United States (23 %), China (20 %), or Netherlands (20 %) (Fig. 1A, Fig. 1B). Most studies were standard peer-reviewed publications (75 %) (Fig. 1A). The range of publication dates included in this study was 2015–2024, with most studies taking place in 2021, 2022, or 2023 (Fig. 1C). The majority of studies did not publicly release data or code (55 %), with only 32 % releasing data, 29 % releasing code, and 16 % releasing both data and code; relative code and data availability increased in 2021, 2022, and 2023 (Fig. 1C).

**Fig. 2.**
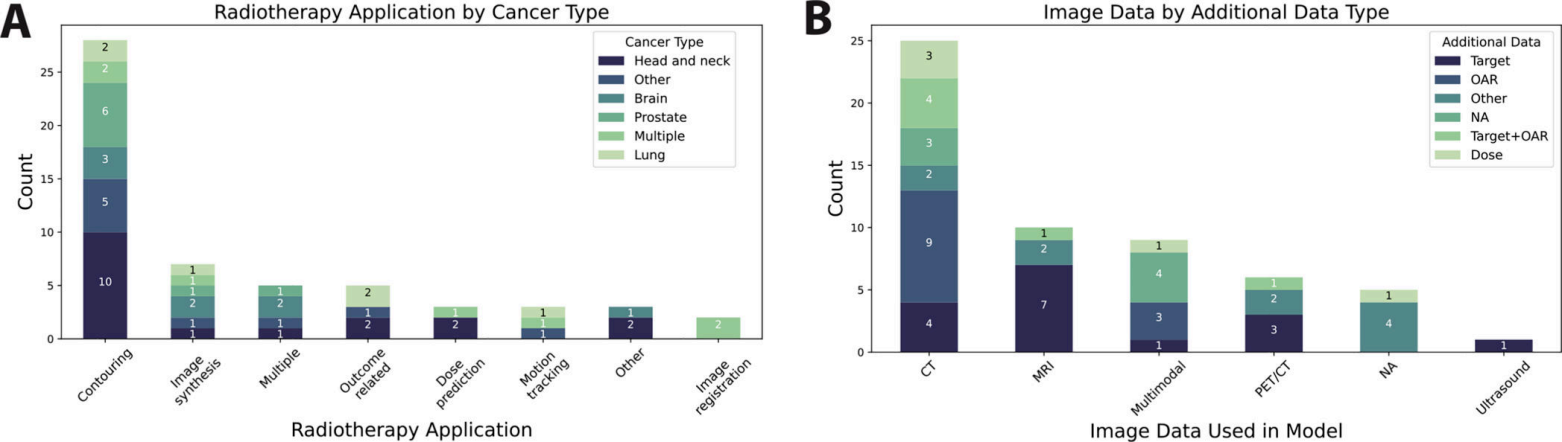
Radiotherapy characteristics. (**A**) Stacked barplot showing cancer disease site per each radiotherapy application domain. “Other” category for cancer type included cervical, liver, esophageal, pancreatic, cardiac, breast, pelvic. “Other” category for radiotherapy application included nodal classification, tumor growth modeling, and image correction. (**B**) Stacked barplot showing additional data per each imaging modality represented. “Other” category for additional data included registration transforms, respiratory trace, K-space, fiducial, clinical data, target + clinical data, dose + clinical data, and dose + clinical data + target + probability map. Each item in the barplots correspond to one study.

Multiple disease sites were included: head and neck, prostate, brain, lung, cervical, liver, esophageal, pancreatic, cardiac, breast, pelvic. Most studies were applied to head and neck cancer patient populations (32 %) (Fig. 2A). Ten RT application domains were involved: contouring, image synthesis, outcome-related, motion tracking, dose planning, image registration, nodal classification, tumor growth modeling, image correction. Most applications were focused on contouring (50 %) (Fig. 2A). Most used medical imaging data in some capacity — 45 % of studies utilized CT data (Fig. 2B); only 9 % did not utilize medical imaging. The majority of studies also utilized target structures (29 %), OARs (21 %), or both (11 %) as input data in their algorithms (Fig. 2B); only 13 % of included studies used RT dose in their algorithms.

**Fig. 3.**
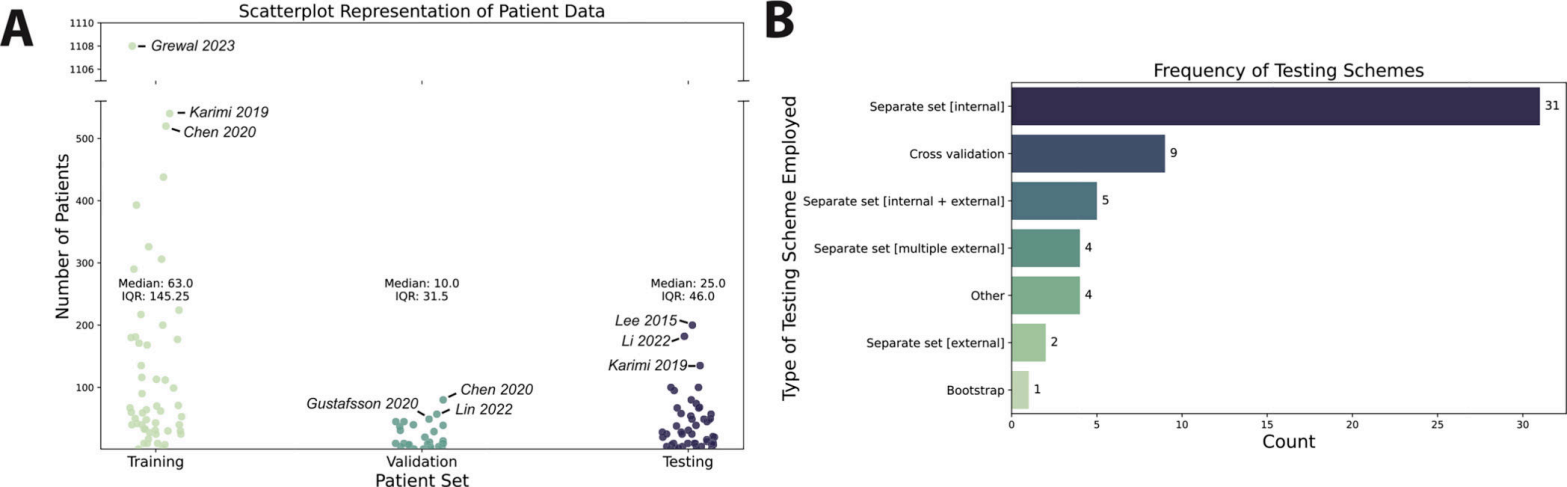
Artificial intelligence characteristics. (**A**) Scatter plot showing number of training, validation, and testing patients used in studies. Only studies that explicitly reported patient-level sample sizes are included. The three studies with the highest sample sizes in each category are annotated. (**B**) Bar plot showing types of testing strategies used in studies. Each item in the barplot corresponds to one study.

The vast majority of the studies (88 %) used labeled data for model training, i.e., supervised learning. Median (interquartile range) patient sample sizes were 63 (145.25), 10 (31.5), and 25 (46) for training, validation, and test datasets (Fig. 3A). Most studies used a separate dataset for model validation (40 %) compared to cross-validation approaches (30 %), while 30 % did not mention their validation methods. Most studies used a separate test set composed of internal, i.e., single source data (55 %); only 7 % of studies used multiple external validation datasets for testing (Fig. 3B).

**Fig. 4.**
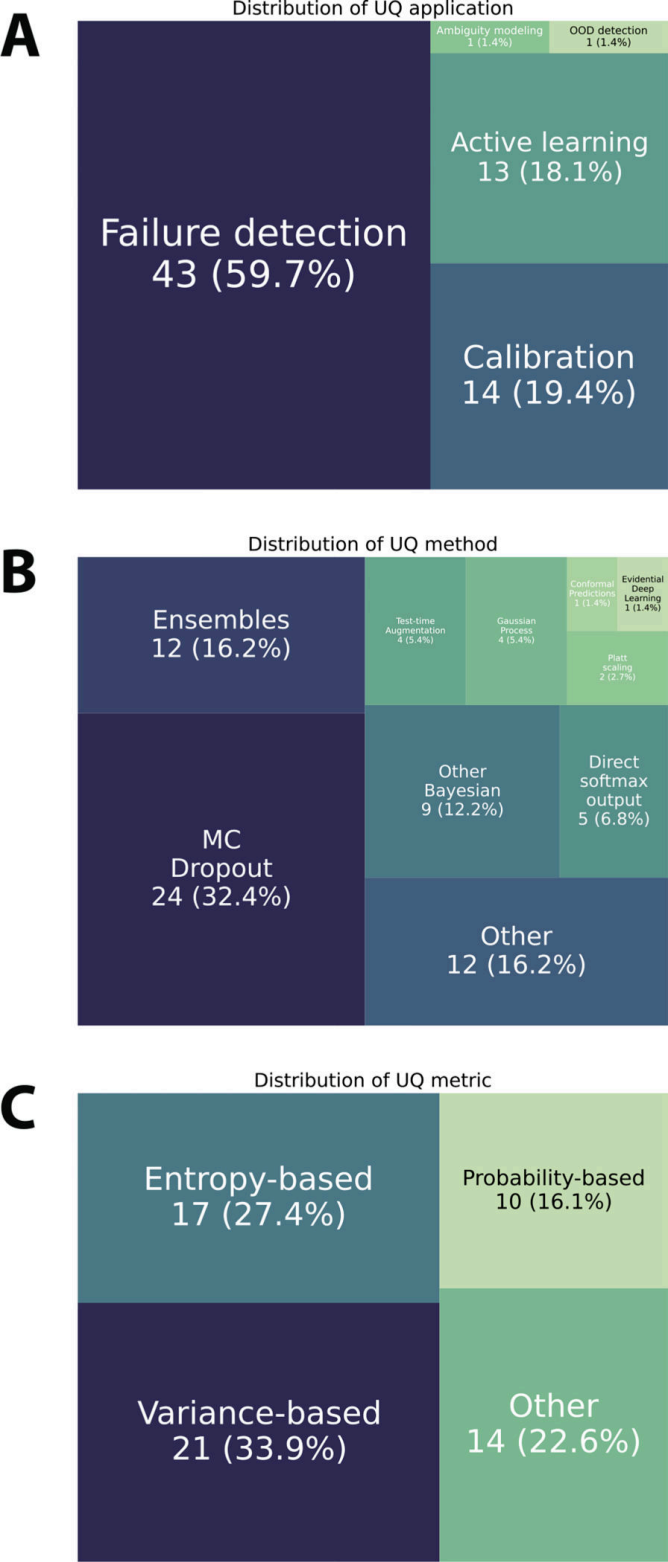
Uncertainty quantification characteristics. (**A**) Tree map of uncertainty quantification applications represented in the studies. (**B**) Tree map of uncertainty quantification methods represented in the studies. (**C**) Tree map of uncertainty quantification metrics represented in the studies. Each item in the tree maps correspond to a reported item (could be multiple per study).

Most studies investigated failure detection applications (60 % of reported applications) followed by calibration (19 %) and active learning (18 %), with only a few studies investigating ambiguity modeling or out-of-distribution detection (Fig. 4A). The majority of studies used MC Dropout (32 % of reported methods), followed by ensembles (16 %) and other methods (16 %), with a smaller number of studies using other Bayesian methods, direct softmax outputs, test-time augmentation, gaussian processes, Platt scaling, conformal prediction, and evidential deep learning (Fig. 4B). In terms of calculated uncertainty metrics, most studies reported using variance-based methods (34 % of reported metrics) and entropy-based metrics (27 %), followed by other self-defined metrics (23 %), with the smallest number reporting probability based metrics (16 %) (Fig. 4C). Most studies did not explicitly report if they investigated aleatoric or epistemic uncertainty (77 % of studies) and used a combination of quantitative and qualitative experiments for investigating uncertainty (52 %). Only 20 % of contouring studies investigating failure detection applications used preferred UQ methods and metrics from the ValUES framework (Appendix D).

Five systematic/scoping review papers related to UQ in medicine were selected for the overlap comparison. Only six studies investigated in these review papers overlapped with our 56 extracted manuscripts (Table 2).

## Discussion

The field of RT is increasingly incorporating AI into its various workflows. Although AI UQ is well-established in computer science, its adaptation to medicine and RT is still in its early stages. Incorporating UQ in AI models used in RT workflows has the potential to increase clinician confidence, helping bridge the translational gap between single institutional model development to multi-institutional clinical implementation. Our scoping review is a pioneering effort to systematically examine the application of UQ concepts within RT.

We identified several trends in UQ research in RT, likely driven by technical innovations within the broader AI research community. Mirroring practices from computer science [38], a considerable number of manuscripts were conference proceedings rather than traditional publications. Notably, we found a predominant contribution of studies from the European Union (EU). Given stringent EU data protection laws — such as The General Data Protection Regulation (GDPR) which poses challenges for secondary data use in research [97] — this raises considerations for how practitioners of UQ in RT should value data sharing. There exists a known tension between open science principles and protecting patient privacy. Although code and data availability have become standard in AI-related research, medical applications lag in this regard [98–100]. Our analysis revealed a gradual increase in code and data availability over time, reflecting a slowly evolving open science ethos in the RT community [20]. Notably, the National Institutes of Health (NIH) new Data Management and Sharing policy, effective January 2023, mandates broader data sharing for NIH-supported research [101], aligning with these open science principles. In light of these findings, we advocate for the publication of code and anonymized data in AI UQ research in RT wherever feasible to enhance reproducibility. When code or data sharing is not possible, future work should encourage privacy-preserving methodologies, like federated learning [102], as viable alternatives to conventional data sharing practices.

The extracted manuscripts covered various RT application spaces and disease sites. Auto-contouring was the most common application, aligning with its prevalence in AI-based RT [10,103,104]. Many studies focused on head and neck cancer, likely due to the complexity of this disease site, which requires precise delineation of numerous organs at risk (OARs) and challenging tumor-related target structures [105]. Most auto-contouring studies investigated OARs on CT imaging. Target structures were often generated with imaging modalities beyond CT, such as MRI for enhanced soft tissue contrast or PET for metabolic activity incorporation, matching physician practice patterns [106]. The variability observed in OARs and target structures can be characterized as aleatoric uncertainty driven by physician judgment [104,107,108]. Interestingly, Karimi et al. [49] showed that reducing aleatoric uncertainty may not be as critical as ensuring large training set sizes, at least in their specific prostate cancer target use-case. This finding suggests that, for institutional model training and fine-tuning, focusing on expanding dataset size could be more impactful in reducing overall model uncertainty rather than minimizing underlying contour variability (i.e., addressing factors associated with epistemic uncertainty), though this should likely be balanced with the known influence of consistent contouring on overall model performance [109]. Of note, it has recently been suggested that RT auto-contouring performance is saturating [110], driving the need for research into additional research spaces such as UQ. Future research may benefit from exploring UQ techniques specifically tailored to address aleatoric uncertainty in auto-contouring models, considering the differences in variability between OARs and target structures.

A distinct facet in RT workflows compared to other oncologic research areas is the presence of multidimensional and complex dosimetric treatment data. DL-based dose prediction is emerging as a promising alternative to traditional knowledge-based planning approaches, offering the potential for improved accuracy, reliability, and efficiency in patient-specific plan optimization [111]. The uncertainty in DL-based dose prediction models could be critical, as it could determine when model-generated plans should be directly accepted, or if manual interventions from physicians and physicists are required to improve plan quality [80]. Surprisingly, in our review there were relatively few manuscripts directly investigating model UQ in dose prediction applications [56,65,72,80,95]. This scarcity is mirrored in outcome prediction research, where only a few studies explored dose-related toxicities [42,70,72,78], as opposed to broader oncologic outcomes like survival. Naturally, a major challenge in outcome-related research stems from the limited availability of training samples. Compared with studies that leverage granular inputs (e.g., multiple image slices representing one patient), dose-related toxicity outcomes often can only be represented at the patient level, which may explain the relative scarcity of literature.

Consistent with similar medical domains reliant on imaging [112], the majority of studies in our review employed supervised learning techniques, which involve training models on labeled data to make predictions based on new, unseen data. A minority of studies explored unsupervised learning approaches [43,57,75,85,95,96] where models learn patterns and relationships from data without explicit labels. In our review, these unsupervised methods were particularly useful for image synthesis tasks. Only one study utilized reinforcement learning [78], a technique where an agent learns to make decisions based on rewards and punishments. Regardless of the ML technique employed, training dataset sizes were generally small, with the three largest patient cohorts corresponding to auto-contouring studies (520–1108 patients) [49,51,86]. ML models often struggle with small sample sizes, especially when considering complex, multidimensional data like medical images, where models must learn intricate generalizable spatial relationships. As previously noted, this challenge is intensified when prediction outputs are restricted to broad patient-level information, such as toxicity or prognosis, with each patient representing a single data point. Notably, tasks that utilize more granular training information, like auto-contouring or image synthesis, can effectively utilize the numerous data points within each image, allowing these models to achieve reasonable performance despite the limited number of patients [110,113,114]. However, given these relatively small patient sample sizes, it is likely that intrinsic epistemic uncertainty would be high. Subsequently, carefully designed UQ may help identify patients for whom the model’s predictions are more reliable. Finally, despite the importance of using diverse and heterogeneous data for uncertainty experiments, particularly for determining how well models handle new and unknown data scenarios [21], only a handful of studies attempted to utilize multiple external test datasets [48,57,88,92]. Interestingly, this was in stark contrast to a previous scoping review on AI UQ in a broader medical context which identified a predominance of external dataset testing [31].

Ideally, UQ methods should be validated across a broad spectrum of downstream uncertainty tasks [21]. However, in our review only the study by Yang et al. [80] explored a comprehensive approach. Focusing on UQ-guided patient-specific RT dose delivery quality assurance using gamma passing rate, they evaluated several UQ applications simultaneously such as active learning, calibration, failure detection, and out-of-distribution detection. Most other studies focused on singular UQ applications, with failure detection by far being the most common. In these studies, UQ is typically used as a screening tool. For instance, Sahlsten et al. evaluated multiple UQ methods and metrics for gross tumor contouring in oropharyngeal cancer, proposing a potential workflow where highly uncertain cases are referred to clinicians for additional manual review [91]. Failure detection’s high prevalence in our review may partially be explained by the fact that it can be accomplished without direct UQ methods (e.g., end-to-end quality assurance models [115]), contributing to its overrepresentation in the general literature. Interestingly, model calibration, which attempts to ensure that predicted probabilities align with observed outcomes, appears somewhat underexplored in the reviewed studies, despite its historical importance in uncertainty discussions [27]. This oversight might stem from an inherent assumption that some UQ model outputs are already calibrated [28], which may not always hold true [29]. It should be emphasized that simple methods of post-hoc calibration, such as performed by Nomura et al. for proton CT image correction [66], could rapidly be applied to most UQ methods. A minority of studies also used uncertainty in active learning frameworks, where the model selects the most informative data points for training based on their uncertainty. For example, Grewal et al. develop a cervical cancer OAR contouring model that incorporates an entropy-guided loss function designed to optimize performance in the presence of missing annotations [86]. Ambiguity modeling and out-of-distribution detection were vastly underrepresented, with only one study each investigating these areas: Li et al. [71] implemented interobserver variability as ground truth uncertainty in auto-contouring, while Yang et al. [80] determined out-of-distribution dose delivery using external datasets. The scarcity of out-of-distribution detection studies in our review may be attributed to the limitations of popular UQ methods like Monte Carlo dropout in reliably identifying out-of-distribution samples, as demonstrated in recent literature [116,117], highlighting a potentially unmet need for more robust UQ techniques tailored to this application in RT.

In line with previous review literature [31,33], Monte Carlo dropout was the most frequently used UQ method in our scoping review. Monte Carlo dropout has gained widespread acceptance for its simplicity and effectiveness as a scalable approach to approximate Bayesian inference. The prevalence of Monte Carlo dropout in our review likely stems from its minimal modification requirements to existing DL architectures, as models implementing routine dropout during training could seamlessly apply dropout during testing. A similar ease of implementation may also explain the widespread use of ensembles. Monte Carlo dropout, and other common methods such as ensembles often yield comparable outputs [118], so the superiority of specific methods is unclear and likely context-specific. It is also likely that certain UQ methods incur greater computational costs during training and/or inference (e.g., Monte Carlo dropout and ensembles require multiple network passes and thus longer runtimes) [119], a factor that must be weighed when evaluating their favorability for practical applications. In terms of uncertainty metrics, the majority of the reviewed studies favored variance-based or entropy-based metrics, aligning with their established prevalence in the literature [31,33]. Notably, the recently proposed ValUES segmentation framework by Kahl et al. identifies optimal UQ methods and metrics [21], but our review reveals that only a small number of contouring studies investigating failure detection implemented these recommended approaches (i.e., use of predictive uncertainty and ensembles).

There appears to be a significant gap in the adoption of newer, innovative UQ methods in RT. For instance, only one study in our review, conducted by Dohopolski et al. on head and neck cancer outcome prediction [70], explored emerging techniques like evidential deep learning and conformal prediction. Evidential deep learning has been suggested to be more computationally efficient and better calibrated than other conventional methods such as Monte Carlo dropout and ensembles [120], so could be favorable where quick reliable UQ estimates would be needed (e.g., online adaptive RT). In a similar vein, conformal prediction can offer quick computationally efficient results and adapts to any underlying AI model [26,121], providing statistically guaranteed uncertainty estimates that could bolster clinician confidence in treatment decisions across a wide range of RT scenarios. Moreover, while qualitative analyses through heatmap visualizations were common in our extracted studies, it has been argued that conventional methods fall short in providing spatially correlated estimates (e.g., UQ estimates would be returned for each pixel independently in a contouring model) [122]. This limitation may hinder these methods’ ultimate clinical utility and drive the development of alternative approaches, such as Stochastic Segmentation Networks, which can generate multiple spatially coherent solutions simultaneously, thereby providing clinicians with a spectrum of plausible options to evaluate [123]. Generally, the adoption of newer and underexplored UQ methods could potentially address limitations of current popular conventional methods and enhance the application of UQ in RT.

Historically, the AI UQ research community has placed significant emphasis on distinguishing between aleatoric and epistemic uncertainty. However, recent literature suggests that the ability to differentiate aleatoric from epistemic uncertainty using popular contemporary UQ techniques may not be as clear-cut as previously thought [21,22]. Our review revealed that the majority of studies surveyed did not explicitly identify whether their models captured aleatoric or epistemic uncertainty. This observation suggests that, at least within the RT community, the distinction between these types of uncertainty may not be deemed critical enough to warrant specific mention. Moreover, the practical significance of distinguishing between epistemic and aleatoric uncertainty may vary depending on the study’s objectives; for instance, if the primary goal is to quickly flag errors for broader human oversight (e.g., failure detection), a detailed separation of these uncertainty types might not be crucial.

Although a principal motivation behind UQ in medical AI is often believed to be the enhancement of clinician trust [4], none of the studies we reviewed explicitly investigated the influence of UQ estimates on end-user trust or decision making. This is particularly interesting given that most studies dealt with failure detection applications which would necessitate a secondary review by a clinician. Literature within diagnostic imaging applications has demonstrated that the presentation of differential outputs from an AI algorithm can impact user performance, confidence, and reliance to varying degrees [124,125]. Similarly, human–machine interaction studies beyond the medical domain have demonstrated that the careful modulation of presented uncertainty can significantly influence user trust in automated systems [126,127]. Examining how UQ influences clinician trust and decision-making in RT through targeted human–machine interaction experiments could further elucidate the real-world impact of these tools, suggesting a vital direction for future research.

To further emphasize the novelty and need of our study, we investigated the intersection between the publications we reported on and those already cited across existing related systematic and scoping review papers. Importantly, our examination revealed just six instances of citation overlap across five distinct review papers, highlighting the originality of our research and a significant gap within the current academic discourse.

Our study, while striving for a structured and comprehensive overview of existing literature, has some limitations. Firstly, the landscape of available studies was largely limited to those indexed in the queried databases, although we supplemented our search with hand-selected literature to ensure broader coverage. Secondly, the emergent field of AI UQ presents challenges in applying traditional study quality assessment guidelines, as tailored guidelines are not yet available. However, we have taken inspiration from existing reporting guidelines, such as TRIPOD [39] and CLAIM [40], to extract relevant modeling information for our review. Furthermore, we have incorporated aspects of the newly proposed ValUES framework which aims to provide a systematic approach to validating uncertainty estimation in semantic segmentation [21], adapting its principles to enrich our review process.

Finally, an important facet of UQ that we have not considered, but should be the focus of future work is related to model bias. Notably, UQ is often part of broader discussions related to AI explainability, which is often more directly related to identifying and addressing bias. Although explainability and bias in medical AI has garnered significant attention [128], investigation of these topics in RT remains limited [16]. Moreover, while a recent small-scale study indicated that geographic biases in RT auto-contouring models are minimal [129], the necessary broader investigations across various applications have yet to be conducted. The potential for perpetuating biases and inequalities escalates when AI models function as “black boxes” with obscured decision-making processes [130]. UQ, potentially in combination with other explainability methods, could ultimately allow for improved bias detection and mitigation [5].

## Conclusions

The escalating use of UQ for RT applications signifies a key shift towards potentially more clinically impactful AI tools. Our scoping review uncovered a broad spectrum of RT applications and disease sites that have implemented UQ. However, we observed a concentration of efforts in specific areas, such as auto-contouring, while crucial domains like dose and outcome prediction were underrepresented. Moreover, although established techniques like Monte Carlo dropout and ensembles were frequently used, the exploration of alternative methods, such as conformal prediction, was limited. Our review also underscored the need to rigorously explore and validate a greater diversity of uncertainty application categories extending beyond failure detection—the most prevalent application in our findings—to include areas such as active learning, ambiguity modeling, calibration, and out-of-distribution detection. Notably, the majority of studies lacked code and dataset sharing suggesting a need for improved transparency and reproducibility in AI UQ research for RT. Additionally, the absence of standardized guidelines for implementing and reporting AI UQ in RT highlights a crucial area for future research. Addressing these gaps by broadening UQ applications, fostering model transparency, and developing comprehensive guidelines could significantly advance UQ in RT research.

## Supplementary Material

MMC1

## Figures and Tables

**Table 1:** Comprehensive listing of final studies analyzed in this scoping review. Data of interest were split into four main categories: general study characteristics, radiotherapy (RT) characteristics, artificial intelligence (AI) characteristics, and uncertainty quantification (UQ) characteristics. Rows are ordered by ascending publication year and study ID. Additional abbreviations: organ at risk = OAR, machine learning = ML, cross validation = CV, failure detection = FD, active learning = AL, ambiguity modeling = AM, out-of-distribution detection = OODD, Gaussian Process = GP, Ensemble = ENS, Other Bayesian = OB, Platt Scaling = PS, MC Dropout = MCD, Test-time Augmentation = TTA, Conformal Prediction = CP, Evidential Deep Learning = EDL, Direct softmax output = DSO.

General Study Characteristics						RT Characteristics				AI Characteristics						UQ Characteristics				

Study ID	Paper type	Year	Location	Data avail.	Code avail.	RT applic.	Image data	Additional data	Cancer type	ML type	Training patients	Valid. patients	Valid. type	Testing patients	Testing type	UQ applic.	UQ type	UQ method	UQ metric	UQ experiment
Bukhari 2015 [41]	Standard pub.	2015	Korea	No	No	Motion tracking	NA	Repiratory trace	Lung	Supervised	31	31	CV	31	Separate set [internal]	FD	Unspecified	GP	Variance-based	Quant.
Lee 2015 [42]	Standard pub.	2015	Canada	No	No	Outcome related	NA	Clinical	Lung	Supervised	53	Unspecified	Unspecified	200	Bootstrap	Calib.	Unspecified	ENS; OB	Other	Quant.
Bragman 2018 [43]	Conf. proc.	2018	UK	No	No	Multiple	Multimodal	OAR	Prostate	Mixed	10	Unspecified	Unspecified	5	Cross validation	FD	Both	MCD; OB	Other	Quant. + Qual.
Jungo 2018a [44]	Conf. proc.	2018	SUI	No	No	Contouring	MRI	Target	Brain	Supervised	25	Unspecified	Unspecified	5	Cross validation	FD	Unspecified	MCD	Entropy-based	Quant. + Qual.
Jungo 2018b [45]	Conf. proc.	2018	SUI	No	No	Contouring	MRI	Target	Brain	Supervised	25	Unspecified	Unspecified	5	Cross validation	FD	Unspecified	MCD; OB	Entropy-based	Quant. + Qual.
Ninomiya 2018 [46]	Conf. proc.	2018	Japan	No	No	Contouring	CT	Target + OAR	Prostate	Supervised	43	Unspecified	Unspecified	1	Cross validation	FD	Unspecified	OB	Probability-based	Quant.
Qin 2018 [47]	Standard pub.	2018	China	No	No	Contouring	CT	OAR	Liver	Supervised	90	Unspecified	Unspecified	10	Cross validation	FD	Unspecified	DSO	Entropy-based	Qual.
Sentker 2018 [48]	Conf. proc.	2018	Germany	Yes	Yes	Image registration	CT	Registration_transforms	Multiple	Supervised	59	Unspecified	Unspecified	16	Separate set [multiple external]	FD	Unspecified	MCD	Variance-based	Quant. + Qual.
Karimi 2019 [49]	Standard pub.	2019	Canada	No	No	Contouring	Ultrasound	Target	Prostate	Supervised	540	Unspecified	Unspecified	135	Cross validation	AL; Calib.	Both	MCD; ENS; PS	Other	Quant. + Qual.
Lipkova 2019 [50]	Standard pub.	2019	Germany	Yes	Yes	Tumor growth modeling	Multimodal	Target	Brain	Supervised	8	Unspecified	Unspecified	8	Other	AL; Calib.	Unspecified	OB	Variance-based	Quant.
Chen 2020 [51]	Standard pub.	2020	China	No	No	Contouring	CT	Target	Breast	Supervised	520	80	Separate set	80	Separate set [internal]	FD	Unspecified	DSO	Other	Quant.
Dohopolski 2020 [52]	Standard pub.	2020	USA	No	No	Nodal classification	PET/CT	Target + OAR	Head and neck	Supervised	Unspecified	Unspecified	Separate set	Unspecified	Separate set [internal]	FD	Both	MCD; TTA	Entropy-based	Quant.
Gustafsson 2020 [53]	Standard pub.	2020	Sweden	Yes	Yes	Contouring	MRI	Fiducial	Prostate	Supervised	326	49	CV	39	Separate set [internal]	FD	Unspecified	DSO	Other	Quant.
Hansch 2020 [54]	Standard pub.	2020	NL	No	No	Contouring	Multimodal	OAR	Brain	Supervised	27	9	Separate set	9	Separate set [internal]	FD	Unspecified	MCD	Entropy-based	Quant. + Qual.
Maspero 2020 [55]	Standard pub.	2020	NL	No	No	Image synthesis	Multimodal	OAR	Brain	Supervised	30	10	CV	20	Separate set [internal]	FD	Unspecified	ENS	Variance-based	Quant. + Qual.
Nomura 2020 [56]	Standard pub.	2020	Japan	Yes	No	Dose prediction	CT	Dose	Head and neck	Supervised	116	39	Separate set	38	Separate set [internal]	FD	Unspecified	Other	Variance-based	Quant. + Qual.
vanHarten 2020 [57]	Conf. proc.	2020	NL	No	No	Image synthesis	Multimodal	NA	Brain	Unsupervised	30	2	Separate set	74	Separate set [multiple external]external]	FD	Unspecified	ENS	Other	Quant. + Qual.
Balagopal 2021 [58]	Standard pub.	2021	USA	No	Yes	Contouring	CT	Target + OAR	Prostate	Supervised	290	29	CV	50	Separate set [internal]	FD	Unspecified	MCD	Variance-based	Quant. + Qual.
Dasgupta 2021 [59]	Standard pub.	2021	Canada	No	No	Multiple	MRI	Target	Brain	Supervised	42	8	CV	49	Separate set [internal + external]	Calib.	Unspecified	Other; PS	Probability-based	Quant.
Diao 2021 [60]	Standard pub.	2021	China	Yes	Yes	Contouring	PET/CT	Target	Multiple*	Supervised	99	14	Separate set	28	Separate set [internal]	AL	Both	Other	Entropy-based	Quant. + Qual.
Kajikawa 2021 [61]	Standard pub.	2021	Japan	No	No	Image synthesis	CT	OAR	Lung*	Supervised	60	12	CV	11	Separate set [internal]	FD	Epistemic	MCD	Variance-based	Quant. + Qual.
Lei 2021 [62]	Standard pub.	2021	China	Yes	Yes	Contouring	CT	OAR	Head and neck	Supervised	177	Unspecified	Unspecified	48	Separate set [internal]	FD	Unspecified	ENS	Entropy-based; Variance-based	Quant. + Qual.
Luo 2021 [63]	Conf.proc.	2021	China	No	Yes	Contouring	MRI	Target	Head and neck	Supervised	180	20	Separate set	58	Separate set [internal]	AL	Unspecified	Other	Other	Quant. + Qual.
Mei 2021 [64]	Standard pub.	2021	China	Yes	Yes	Contouring	CT	Target	Head and neck	Supervised	40	Unspecified	Unspecified	10	Separate set [internal]	FD	Unspecified	ENS	Entropy-based; Variance-based	Quant. + Qual.
Nguyen 2021 [65]	Standard pub.	2021	USA	Yes	No	Dose prediction	CT	Dose	Head and neck	Supervised	200	40	Separate set	100	Separate set [internal]	FD	Unspecified	MCD; ENS	Variance-based	Quant. + Qual.
Nomura 2021 [66]	Standard pub.	2021	USA	Yes	No	Image correction	CT	NA	Head and neck*	Supervised	3	1	Separate set	1	Separate set [internal]	FD; Calib.	Both	ENS; Other	Other	Quant.
Remy 2021 [67]	Standard pub.	2021	Canada	No	No	Motion tracking	MRI	Target	Multiple*	Supervised	10	Unspecified	Unspecified	10	Other	FD	Unspecified	OB	Variance-based	Quant.
vanRooij 2021 [68]	Standard pub.	2021	NL	No	No	Contouring	CT	OAR	Head and neck	Supervised	Unspecified	Unspecified	Separate set	Unspecified	Separate set [internal]	FD; Calib.	Unspecified	MCD	Probability-based	Quant. + Qual.
Zhang 2021 [69]	Standard pub.	2021	China	Yes	No	Contouring	CT	Target + OAR	Lung	Supervised	48	10	CV	28	Separate set [internal + external]	AL	Unspecified	MCD	Other	Qual.
Dohopolski 2022 [70]	Preprint	2022	USA	No	Yes	Outcome related	CT	Dose	Head and neck	Supervised	217	Unspecified	CV	54	Separate set [internal]	FD	Both	MCD; TTA; CP; EDL	Entropy-based; Other	Quant.
Li 2022a [71]	Standard pub.	2022	USA	No	No	Contouring	CT	Target	Prostate	Supervised	306	1	Separate set	3	Separate set [internal]	AL; AM	Unspecified	Other	Other	Qual.
Li 2022b [72]	Standard pub.	2022	USA	No	No	Multiple	NA	Clinical	Liver	Supervised	NA	NA	Separate set	182	Separate set [external]	AL	Unspecified	GP	Probability-based	Quant.
Lin 2022 [73]	Standard pub.	2022	China	No	No	Outcome related	CT	Target + Clinical	Esophageal	Supervised	171	57	Separate set	57	Separate set [internal]	AL	Unspecified	Other	Other	Quant.
Liu 2022 [74]	Standard pub.	2022	China	Yes	No	Contouring	CT	Target + OAR	Pancreatic	Supervised	62	Unspecified	Unspecified	21	Cross validation	AL	Unspecified	MCD	Other	Qual.
Lyu 2022 [75]	Preprint	2022	USA	Yes	No	Image synthesis	Multimodal	NA	Multiple	Unsupervised	17	Unspecified	Unspecified	2	Separate set [internal]	FD	Unspecified	Other	Variance-based	Quant. + Qual.
Mody 2022a [76]	Conf. proc.	2022	NL	Yes	Yes	Contouring	CT	OAR	Head and neck	Supervised	33	Unspecified	Unspecified	25	Separate set [internal + external]	FD; Calib.	Unspecified	MCD; Other	Entropy-based	Quant. + Qual.
Mody 2022b [77]	Conf. proc.	2022	NL	Yes	Yes	Contouring	CT	OAR	Head and neck	Supervised	33	5	Separate set	25	Separate set [internal + external]	FD; Calib.	Both	OB	Entropy-based	Quant.
Sun 2022 [78]	Standard pub.	2022	USA	No	No	Outcome related	NA	Dose + Clinical	Lung	Reinforcement	67	Unspecified	Unspecified	67	Other	FD; AL; Calib.	Unspecified	GP	Variance-based	Quant.
Wang 2022 [79]	Standard pub.	2022	USA	No	Yes	Outcome related	PET/CT	Target + Clinical	Head and neck	Supervised	135	45	CV	45	Cross validation	FD	Both	TTA; Other	Entropy-based; Probability based	Quant.
Yang 2022 [80]	Standard pub.	2022	China	No	No	Dose prediction	NA	Dose	Multiple*	Supervised	Unspecified	Unspecified	Separate set	Unspecified	Separate set [internal + external]	AL; Calib.; FD; OODD	Both	MCD	Entropy-based	Quant.
Zabihollahy 2022 [81]	Standard pub.	2022	USA	No	No	Contouring	MRI	Target + OAR	Cervical	Supervised	112	Unspecified	Separate set	13	Separate set [internal]	FD	Unspecified	MCD	Variance-based	Qual.
Cubero 2023 [82]	Conf. proc.	2023	Spain	No	No	Contouring	CT	OAR	Head and neck	Supervised	40	Unspecified	CV	8	Separate set [internal]	FD	Unspecified	MCD	Entropy-based	Quant. + Qual.
DeBiase 2023 [83]	Standard pub.	2023	NL	No	No	Contouring	PET/CT	Target	Head and neck	Supervised	113	37	CV	25	Separate set [internal]	FD	Unspecified	ENS	Probability-based	Quant.
Ebadi 2023 [84]	Standard pub.	2023	USA	Yes	Yes	Contouring	CT	Target	Lung	Supervised	438	Unspecified	CV	3	Cross validation	FD; Calib.	Unspecified	MCD; ENS	Entropy-based; Variance-based	Quant. + Qual.
Galapon 2023 [85]	Standard pub.	2023	NL	No	No	Image synthesis	Multimodal	NA	Head and neck	Unsupervised	71	10	Separate set	20	Separate set [internal]	FD	Unspecified	MCD	Variance-based	Quant. + Qual.
Grewal 2023 [86]	Conf. proc.	2023	NL	No	Yes	Contouring	CT	OAR	Cervical	Supervised	1108	Unspecified	CV	95	Separate set [internal]	AL	Epistemic	Other	Entropy-based	Quant.
Huttinga 2023 [87]	Standard pub.	2023	NL	No	No	Motion tracking	MRI	K-space	Cardiac*	Supervised	1	Unspecified	Unspecified	1	Other	FD	Unspecified	GP	Probability-based	Quant.
Luan 2023 [88]	Standard pub.	2023	China	Yes	No	Contouring	CT	OAR	Head and neck	Supervised	70	Unspecified	CV	68	Separate set [multiple external]	AL	Unspecified	DSO	Probability-based	Quant. + Qual.
Min 2023 [89]	Standard pub.	2023	AU	No	No	Contouring	MRI	Target	Prostate	Supervised	393	5	Separate set	49	Separate set [internal]	FD	Unspecified	MCD	Variance-based	Quant. + Qual.
Outeiral 2023 [90]	Standard pub.	2023	NL	No	Yes	Contouring	MRI	Target	Multiple	Supervised	181	Unspecified	Separate set	Unspecified	Separate set [internal]	FD	Unspecified	DSO	Probability-based	Quant. + Qual.
Sahlsten 2023 [91]	Preprint	2023	USA	Yes	No	Contouring	PET/CT	Target	Head and neck	Supervised	224	45	CV	67	Separate set [external]	FD	Both	MCD; ENS	Entropy-based; Variance-based	Quant. + Qual.
Smolders 2023 [92]	Standard pub.	2023	SUI	No	Yes	Image registration	CT	NA	Multiple	Mixed	50	10	Separate set	10	Separate set [multiple external]	FD; Calib.	Unspecified	OB; Other	Variance-based	Quant. + Qual.
Tian 2023 [93]	Standard pub.	2023	Germany	Yes	No	Image synthesis	Multimodal	NA	Pelvic	Supervised	10	4	Separate set	5	Separate set [internal]	FD	Unspecified	MCD	Variance-based	Quant.
DeBiase 2024 [94]	Standard pub.	2024	NL	No	No	Multiple	PET/CT	Dose + Clinical + Target + Probability map	Head and neck	Supervised	168	Unspecified	CV	100	Separate set [internal]	FD; Calib.	Unspecified	ENS	Probability-based	Quant.
Li 2024 [95]	Standard pub.	2024	SUI	No	No	Multiple	Multimodal	Dose	Brain	Unsupervised	64	8	CV	10	Separate set [internal]	FD	Unspecified	OB	Other	Quant. + Qual.
Rusanov 2024 [96]	Standard pub.	2024	AU	No	No	Image synthesis	CT	NA	Prostate	Unsupervised	40	5	Separate set	5	Separate set [internal]	FD; Calib.	Both	MCD; TTA	Variance-based	Quant. + Qual.

* Dataset specific notes. Nomura 2021 – phantom replicas derived from human head and neck patient data. Remy 2021 – volunteer data but with specific application to radiotherapy applications. Diao 2021 – utilized two disease sites (soft tissue sarcoma and lymphoma) but combined data into one dataset. Kajikawa 2021 – patients had primary diseases that were not cancer but application of study was specific to radiotherapy. Yang 2022 – primary study involved glioma, lung, and liver cancer patients, out-of-distribution experiments involved breast, cervical, esophageal, tongue, and lung cancer patients. Huttinga 2023 – in vivo studies using volunteers and a ventricular tachycardia patient who received radioablation.

**Table 2:** Study overlap with previous systematic and scoping reviews. Papers contained in previous systematic/scoping reviews related to uncertainty quantification in medicine were compared with papers extracted for our scoping review. Only final papers that were used for data extraction were compared.

Review Paper	Overlapping Citations/Total Citations (%)	Specific Overlapping Manuscript(s)

Zou et al. (2023) [32]	3/56 (5 %)	Jungo et al. (2018) [44], Jungo et al. (2018) [45], Bragman et al. (2018) [43]
Kurz et al. (2022) [33]	0/22 (0 %)	None
Lambert et al. (2024) [34]	4/217 (2 %)	Jungo et al. (2018) [44], Jungo et al. (2018) [45], Balagopal et al. (2021) [58], Mei et al. (2021) [64]
Loftus et al. (2022) [31]	0/30 (0 %)	None
Seoni et al. (2023) [25]	2/144 (1 %)	Jungo et al. (2018) [45], Lipkova et al. (2019) [50]
