# Supplementary material for: Artificial intelligence uncertainty quantification in radiotherapy applications — A scoping review
Source: Radiother Oncol. Author manuscript; Available in PMC 2024 Dec 16. (PMC11648575; doi:10.1016/j.radonc.2024.110542)
Supplement: MMC1 [file NIHMS2025445-supplement-MMC1.docx]

## Appendix A: Additional Figures


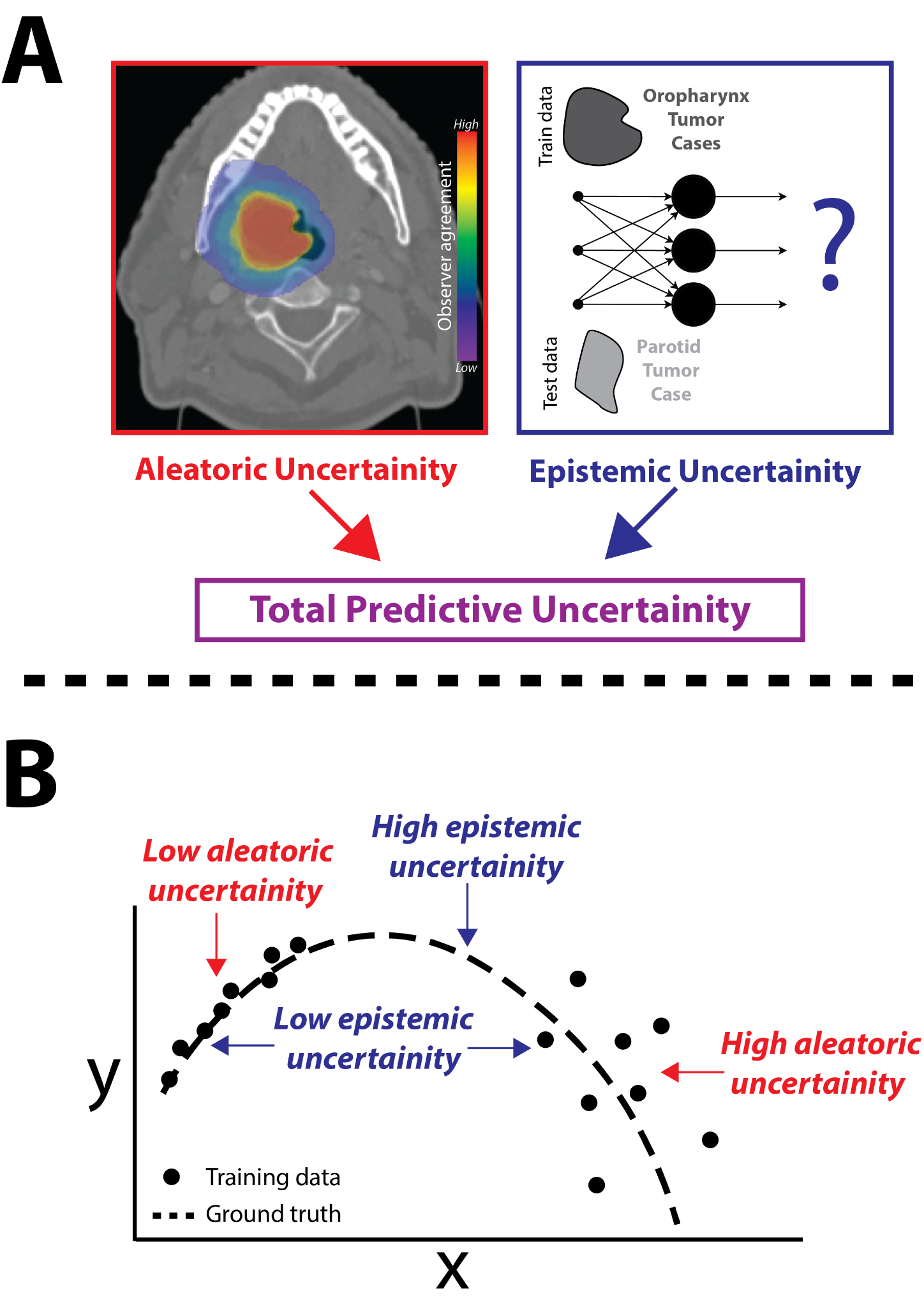


**Figure A1.** Illustrative examples of aleatoric and epistemic uncertainty concepts. (**A**) Left: A computed tomography image of an oropharyngeal cancer patient, overlaid with a probability map of interobserver agreement, illustrates aleatoric uncertainty in segmentation. Example data derived from expert contours from the Contouring Collaborative in Radiation Oncology (doi: 10.1038/s41597-023-02062-w). Right: A hypothetical tumor contouring model trained using oropharyngeal cancer cases would yield high epistemic uncertainty when presented with a parotid tumor case as a byproduct of insufficient training data. The combination of aleatoric and epistemic uncertainties contributes to the total predictive uncertainty. (**B**) A scatterplot of hypothetical variables x and y demonstrates high aleatoric uncertainty in regions with noisy data points and high epistemic uncertainty in regions with sparse data points.


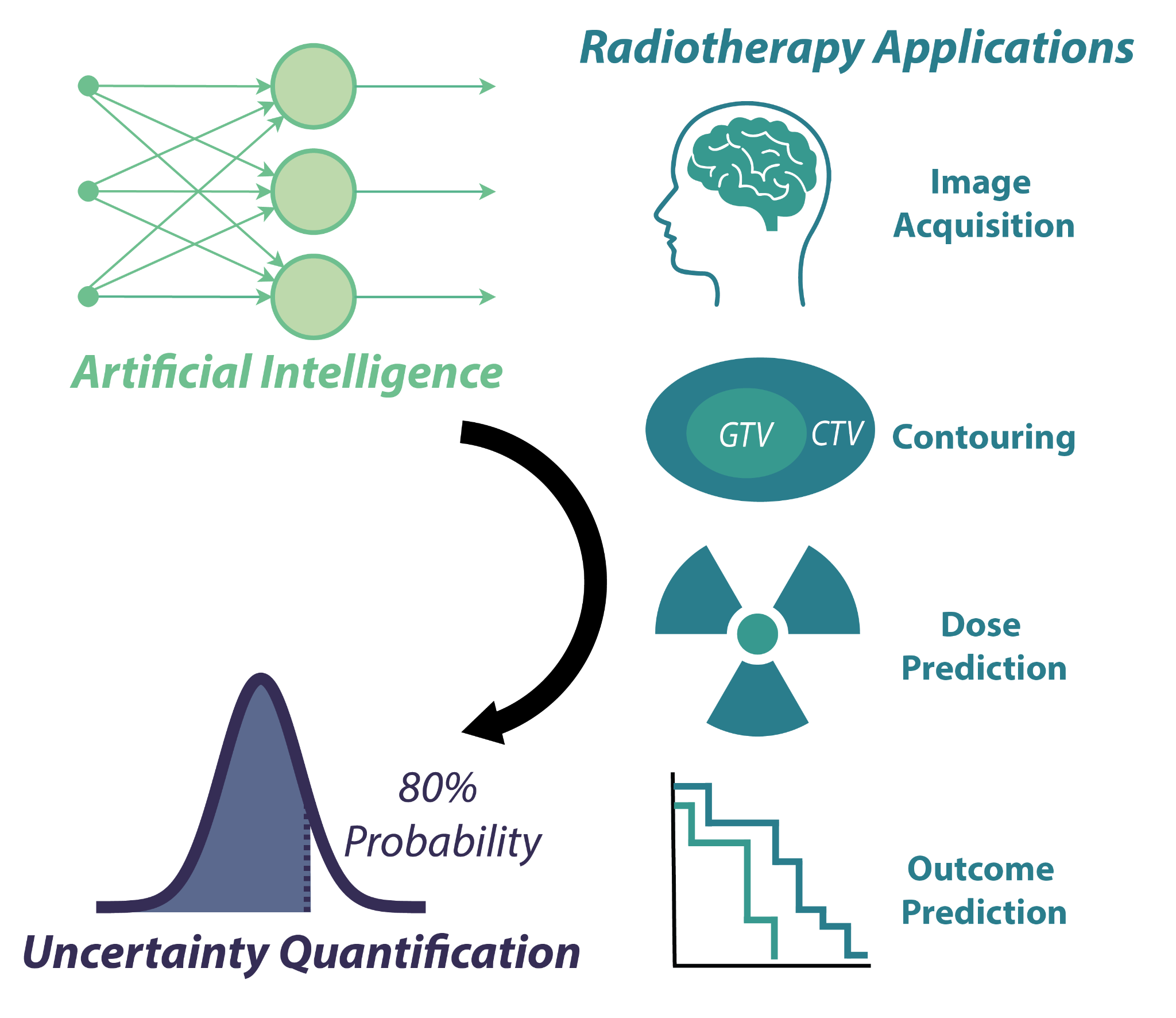


**Figure A2.** Study overview. This scoping review aims to comprehensively evaluate the literature on artificial intelligence models designed to quantify model uncertainty, specifically within the context of radiotherapy applications such as image acquisition, contouring, dose prediction, and outcome prediction, among others.


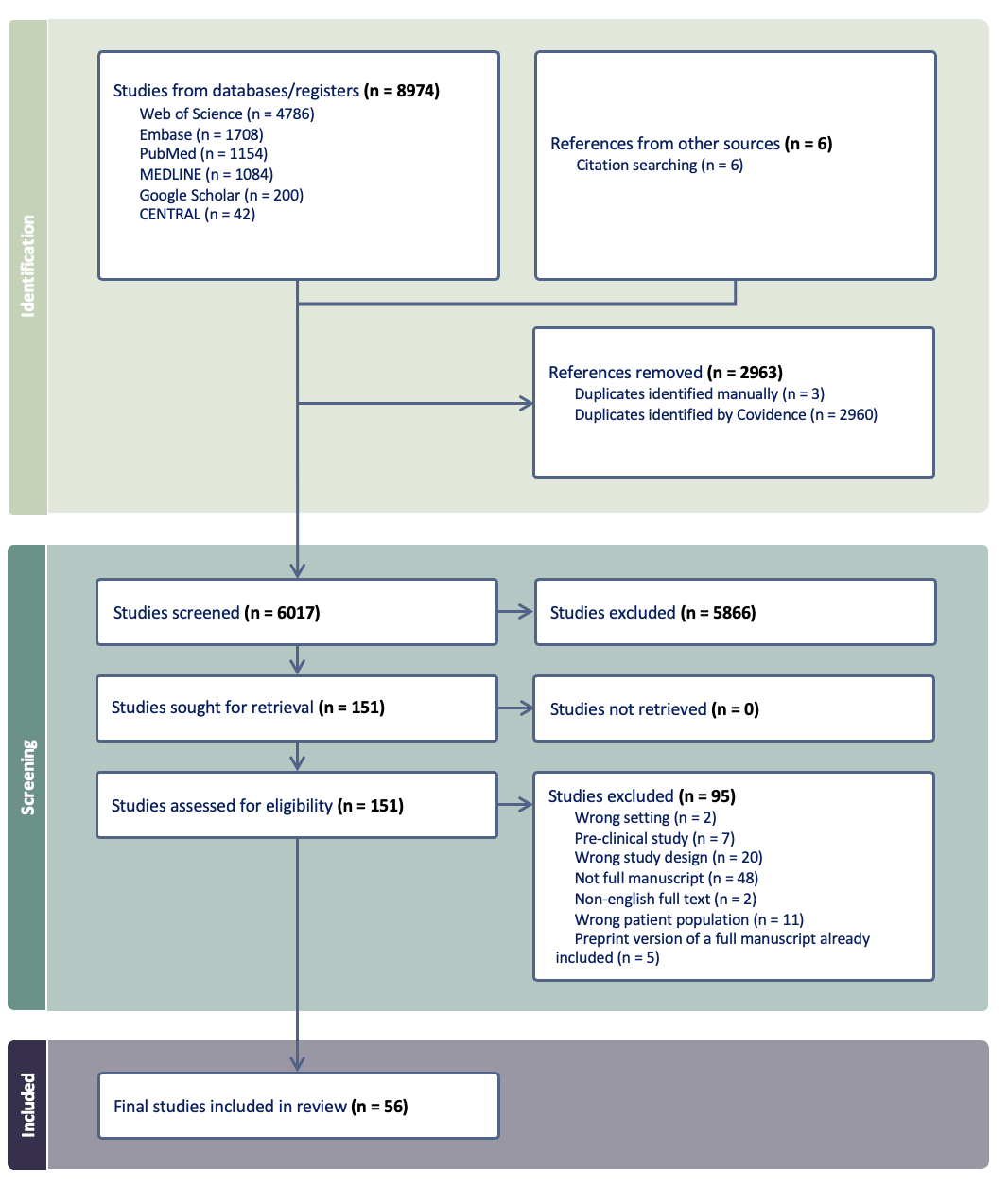


**Figure A3.** Preferred Reporting Items for Systematic Reviews and Meta-Analyses diagram illustrating systematic screening of identified studies. Ultimately, 56 studies out of the initially identified 8980 were included for the final analysis.

## Appendix B: Additional information on manuscript screening and data extraction

### Additional manuscript screening details

Two rounds of screening were performed by all reviewers which took into account all the inclusion criteria listed below. The initial screening (first round) was intended as a quick filtering process based on titles and abstracts to reduce the number of manuscripts into a workable size for eventual full-text screening (second round). Two reviewers (K.A.W. and Z.Y.K.) performed the screening process through Covidence which allows for rapid categorization of articles into inclusion/exclusion piles. Any disagreements were automatically flagged for additional review. Flagged cases underwent further scrutiny via virtual video meetings, where the two initial reviewers were joined by an independent, senior third reviewer (M.J.D.) to meticulously evaluate the contentious manuscripts. This collaborative review culminated in a final vote to decide whether the article merited inclusion. This process was repeated both for initial screening and full-text screening.

Population, concept, context (PCC) criteria for study inclusion:

1. *Population - Human patients undergoing radiotherapy for cancer treatment.* The study should explicitly mention that human patients that were actively undergoing radiotherapy, had plans to undergo radiotherapy, or had already completed radiotherapy were the subjects from which data were derived from. Studies using only preclinical samples (e.g., cell line, animal model, phantom studies, synthetic data) were excluded even if results could eventually be extrapolated to human patients. In rare circumstances where human and preclinical data was combined (e.g., mixed human data and phantom data), these studies were included. Moreover, in rare circumstances data derived from non-cancer patients were included only if directly applicable for radiotherapy specific indications (e.g., radioablation treatment of arrhythmia).
2. *Concept - Utilization of artificial intelligence and uncertainty quantification.* The study should explicitly mention that the underlying modeling technique is related to artificial intelligence or machine learning (e.g., deep learning related or more traditional methods), and must provide a method to quantify the uncertainty or confidence of the underlying model. Ideally, studies should explicitly list training and testing sample sizes, but this was not a strict requirement, particularly for older studies where this stratification was not yet standard. Studies only investigating underlying uncertainties of a radiotherapy related process (e.g., proton range uncertainty, segmentation interobserver variability, etc.) without any indication of a method to quantify predictive model uncertainty were not included.
3. *Context - Radiotherapy applications.* The study should explicitly mention that radiotherapy is the target application domain of the study or belong to a predefined list of radiotherapy applications recognized by the authors (image synthesis, image registration, contouring, dose prediction, outcome prediction). Studies in other related but distinct medical application domains (e.g., diagnostic radiology, interventional radiology, surgical oncology, medical oncology) were excluded unless the study investigated multiple applications within the same paper (e.g., diagnostic radiology applications AND radiotherapy-related applications).

Additional criteria for study inclusion:

1. Full text must be accessible to screeners. Conference abstracts must be linked to a full text (e.g., conference proceeding) or were excluded from the search. Editorials, review papers, and graduate theses were also excluded.
2. Full text must be available in written English, so that it could be appropriately evaluated by all screeners.

### Additional data extraction details

Two human extractors worked in parallel to manually extract data from the final manuscripts. Specific extraction items are detailed below. These items were initially presented as a Covidence template that was used in the data extraction process and then refined if needed to fit into categorical values. All extractions were cross-checked by both reviewers (K.A.W., Z.Y.K.) and a final third reviewer (M.J.D.) when disagreements were found. Extracted data was transformed into machine readable format after initial collection based on agreement between reviewers using a version-controlled online Google Sheets document.

*General Study Characteristics*

1. Manuscript type - If the manuscript is a standard publication (i.e., published in a peer-reviewed journal), a conference proceeding (could be peer-reviewed or not), or a preprint. Articles extracted from preprint servers (e.g., arXiv) would be considered conference proceedings if explicitly indicated in the uploaded document (e.g., *this paper has been accepted to X conference*) and/or a corresponding entry was found on the conference website.
2. Publication year - Year of manuscript upload (in case of preprint) or year of publication as reported by publisher (in case of standard publication or conference proceeding).
3. Geographic location of the study authors - Which country the authors were from as determined from author affiliation information. If not all authors were from the same country, the following hierarchy was used. 1. Country where the majority of authors were from, 2. In the unlikely event of a tie, the country of the corresponding author was reported.
4. Code/data availability - If code and/or data were made publicly available. Relevant datasets DOIs and GitHub URLs were collected and reported where applicable.

*RT Characteristics*

1. Radiotherapy application space - What specific end use the manuscript is developing an artificial intelligence model for? Initially was collected with a free text option but was condensed into a categorical variable with the following possible values: dose planning, image correction, image registration, image synthesis, motion tracking, nodal classification, outcome related, contouring.
2. Specific data types used - What input data is being used for the artificial intelligence models? Initially was collected with a free text option but was condensed into two categorical variables with the following possible values:
   1. Image data: CT, MRI, Multimodal, PET/CT, ultrasound, NA (i.e., none).
   2. Additional data: Clinical, dose, dose+clinical, dose+clinical+target+probability map, fiducial, K-space, organ at risk, registration transforms, respiratory trace, target, target+clinical, target + organ at risk, NA (i.e., none)
3. Cancer type of patients in the study - What were the underlying diagnoses of the patients used in the study? Initially was collected with a free text option but was condensed into a categorical variable with the following possible values: brain, breast, cardiac, cervical, esophageal, head and neck, liver, lung, multiple, pancreatic, pelvic, prostate.

*AI Characteristics*

1. Algorithmic approach - What type of underlying algorithm was used in the study? Initially was collected with a free text option but was condensed into a separate free-text variable and a categorical variable with the following possible values:
   1. Machine learning type: Which overarching domain of machine learning the algorithm is categorized as: supervised, unsupervised, reinforcement, or mixed.
2. Training/validation/testing sample sizes - Specific numbers of training, validation, and testing datapoints used in the study. Data is extracted at most granular level (e.g., some algorithms use axial slices or images as input) and at the patient level. Could be NA if this information was not reported in the manuscript. We chose to focus on patient level data for reported data in our review since it was more clinically relevant and easier to compare between studies.
3. Characteristics of the validation/testing sets - How authors utilized validation and testing sets. Best practices often require separate hold-out sets but this may not always be feasible given dataset constraints. Initially was collected with a free text option but was condensed into two categorical variables with the following possible values:
   1. Validation type: Cross-validation, not specified, separate set.
   2. Testing type: Bootstrap, cross-validation, separate set [external], separate set [internal + external], separate set [internal], separate set [multiple external], other.

*Uncertainty Quantification Characteristics*

1. Uncertainty application category - What is the general use-case of the uncertainty methodology applied? Categories were adapted from existing literature (Kahl et al., [doi: 10.48550/arXiv.2401.08501], Lambert et al. [doi: 10.48550/arXiv.2210.03736]). Studies could investigate multiple applications simultaneously. The following specific categories were utilized:
   1. Active learning: Utilization of uncertainty estimates for improving the model training process.
   2. Ambiguity modeling: Comparison of model uncertainty estimates to a ground truth measure of uncertainty. For example, in segmentation, this could refer to computing the normalized cross-correlation or the generalized energy distance between the pixel-wise model measures and pixel-wise ground truth probability measures.
   3. Calibration: Measurement of agreement between model estimated probabilities and true underlying data distribution probabilities. Popular methods of measuring calibration would include the Expected Calibration Error and the Brier score.
   4. Failure detection: Utilize numerical model uncertainty to determine which cases should be flagged for further inspection. For example, in a segmentation framework the uncertainty estimate could be correlated to a geometric value (e.g., DSC) and subsequently binarized to classify samples below and above an expected correlated geometric value. Related to Misclassification Detection Protocol and Rejection Protocol in Lambert et al. (doi: 10.48550/arXiv.2210.03736).
   5. Out-of-distribution detection: Conceptually similar to failure detection in that an uncertainty measure is used to flag cases. Typically requires a priori identification of in-distribution and out-of-distribution properties for samples (e.g., normal and abnormal images). Typically requires multiple external (out-of-distribution) datasets to implement.
2. Type of uncertainty quantification method used - Specific approach to calculate model uncertainty. Initially was collected with a free text option but was condensed into a single categorical variable. Studies could investigate multiple applications simultaneously. The following specific categories were utilized: Monte Carlo Dropout, Ensembles, Direct Softmax Output, Gaussian Process, Test-time Augmentation, Conformal Prediction, Evidential Deep Learning, Other Bayesian (an explicitly defined Bayesian approach that did not fall into a previous category), Other (bespoke approach developed in a specific paper that did not fall into a previous category).
3. Metrics used for UQ experiments - Any numerical indicators used in the computation of model uncertainty. Initially was collected with a free text option but was condensed into a single categorical variable. Studies could utilize multiple metrics simultaneously. The following specific categories were utilized: Entropy-based, Variance-based, Other (bespoke approach developed in a specific paper).
4. Self-described uncertainty type studied - Whether the study explicitly mentioned they were investigated epistemic and/or aleatoric uncertainty. Possible values of epistemic, aleatoric, both, or unspecified. Only explicit mentions of these terms (or related terms homoscedastic uncertainty and heteroscedastic uncertainty) in the manuscript were considered, otherwise this variable was labeled as unspecified (i.e., no inference about methods was performed on our part).
5. Utilization of quantitative and/or qualitative methods - Whether a uncertainty was presented in a quantitative and/or qualitative manner. Examples of qualitative experiments would include visualizing heatmap pixel-wise representations of model uncertainty in a segmentation problem.

## Appendix C: Additional information on database search criteria

A medical research librarian (D.P.F.) searched MEDLINE (Ovid), Embase (Ovid), PubMed (NLM), Cochrane Library (Wiley), and Web of Science Core Collection (Clarivate) from inception to November 17, 2023, with the search executed on November 20, 2023. A supplementary search of Web of Science Preprint Citation Index (Clarivate) and Google Scholar (Alphabet Inc.) from inception to December 12, 2023 was executed on December 13, 2023 in order to adequately query gray literature such as preprints and conference proceedings. After consultation with the research team, the librarian developed and tailored the search strategy to each database and selected controlled vocabulary (MeSH and Emtree) and natural language terms for the concepts of AI, UQ, and RT. No language, publication date, or other limiters or published search hedges were used.

### Ovid MEDLINE (R) ALL 1946 to November 17, 2023

| **#** | **Searches** | **Results** |
| --- | --- | --- |
| 1 | exp Artificial Intelligence/ | 183179 |
| 2 | ((artificial or machine or deep) adj (learning or intelligence)).ti,ab. | 150066 |
| 3 | ("neural net*" or "support vector" or "decision tree" or "random forest" or "gradient boost*" or bagging or ensemble or radiom*).ti,ab. | 198847 |
| 4 | or/1-3 [AI] | 370737 |
| 5 | Uncertainty/ | 18287 |
| 6 | (uncertain* or aleatoric or epistemic or "monte carlo*" or dropout or Bayes* or "conformal prediction" or "variational inference" or "temperature scaling" or platt or entropy).ti,ab. | 400538 |
| 7 | or/5-6 [Uncertainty] | 404310 |
| 8 | 4 and 7 [AI + Uncertainty] | 24799 |
| 9 | exp Radiotherapy/ | 208974 |
| 10 | exp Radiotherapy Planning, Computer-Assisted/ | 25613 |
| 11 | exp Radiation Oncology/ | 5869 |
| 12 | (radiotherap* or "radio-therap*" or irradiat* or radiat* or chemoradi* or radiochemo* or "chemo-radi*" or "radio-chemo*" or "intensity modulated" or IMRT or EBRT or photon* or proton* or radiosurgery or "radio-surgery" or brachytherapy or "brachy-therapy").ti,ab. | 1086055 |
| 13 | or/9-12 [Radiotherapy] | 1129582 |
| 14 | 8 and 13 [AI + Uncertainty + Radiotherapy] | 1084 |

### Ovid Embase Classic+Embase 1947 to 2023 November 17

| **#** | **Searches** | **Results** |
| --- | --- | --- |
| 1 | exp Artificial Intelligence/ | 89799 |
| 2 | ((artificial or machine or deep) adj (learning or intelligence)).ti,ab. | 176472 |
| 3 | ("neural net*" or "support vector" or "decision tree" or "random forest" or "gradient boost*" or bagging or ensemble or radiom*).ti,ab. | 234393 |
| 4 | or/1-3 [AI] | 385870 |
| 5 | Uncertainty/ | 50932 |
| 6 | (uncertain* or aleatoric or epistemic or "monte carlo*" or dropout or Bayes* or "conformal prediction" or "variational inference" or "temperature scaling" or platt or entropy).ti,ab. | 483767 |
| 7 | or/5-6 [Uncertainty] | 489765 |
| 8 | 4 and 7 [AI + Uncertainty] | 27219 |
| 9 | exp Radiotherapy/ | 736137 |
| 10 | Radiation Oncology/ | 7613 |
| 11 | (radiotherap* or "radio-therap*" or irradiat* or radiat* or chemoradi* or radiochemo* or "chemo-radi*" or "radio-chemo*" or "intensity modulated" or IMRT or EBRT or photon* or proton* or radiosurgery or "radio-surgery" or brachytherapy or "brachy-therapy").ti,ab. | 1462005 |
| 12 | or/9-11 [Radiotherapy] | 1652692 |
| 13 | 8 and 12 [AI + Uncertainty + Radiotherapy] | 1708 |

### PubMed (NLM)

(("artificial intelligence"[MeSH Terms] OR "artificial learning"[Title/Abstract] OR "artificial intelligence"[Title/Abstract] OR "machine learning"[Title/Abstract] OR "machine intelligence"[Title/Abstract] OR "deep learning"[Title/Abstract] OR "deep intelligence"[Title/Abstract] OR "neural net*"[Title/Abstract] OR "support vector"[Title/Abstract] OR "decision tree"[Title/Abstract] OR "random forest"[Title/Abstract] OR "gradient boost*"[Title/Abstract] OR "bagging"[Title/Abstract] OR "ensemble"[Title/Abstract] OR "radiom*"[Title/Abstract])

AND

("uncertainty"[MeSH Terms] OR "uncertain*"[Title/Abstract] OR "aleatoric"[Title/Abstract] OR "epistemic"[Title/Abstract] OR "monte carlo*"[Title/Abstract] OR "dropout"[Title/Abstract] OR "bayes*"[Title/Abstract] OR "conformal prediction"[Title/Abstract] OR "variational inference"[Title/Abstract] OR "temperature scaling"[Title/Abstract] OR "platt"[Title/Abstract] OR "entropy"[Title/Abstract])

AND

("radiotherapy"[MeSH Terms] OR "radiotherapy planning, computer assisted"[MeSH Terms] OR "radiation oncology"[MeSH Terms] OR "radiotherap*"[Title/Abstract] OR "radio therap*"[Title/Abstract] OR "irradiat*"[Title/Abstract] OR "radiat*"[Title/Abstract] OR "chemoradi*"[Title/Abstract] OR "radiochemo*"[Title/Abstract] OR "chemo radi*"[Title/Abstract] OR "radio chemo*"[Title/Abstract] OR "intensity modulated"[Title/Abstract] OR "IMRT"[Title/Abstract] OR "EBRT"[Title/Abstract] OR "photon*"[Title/Abstract] OR "proton*"[Title/Abstract] OR "radiosurgery"[Title/Abstract] OR "radio-surgery"[Title/Abstract] OR "brachytherapy"[Title/Abstract] OR "brachy-therapy"[Title/Abstract])) Results = 1154

### Cochrane Library (Wiley)

| ID | Search | Results |
| --- | --- | --- |
| #1 | MeSH descriptor: [Artificial Intelligence] explode all trees | 2958 |
| #2 | ("artificial learning" or "artificial intelligence" or "machine learning" or "machine intelligence" or "deep learning" or "deep intelligence" or "support vector" or "decision tree" or "random forest" or bagging or ensemble or radiom*):ti,ab or ((neural NEXT net*) or (gradient NEXT boost*)):ti,ab | 7392 |
| #3 | {or #1-#2} | 8923 |
| #4 | MeSH descriptor: [Uncertainty] explode all trees | 421 |
| #5 | (uncertain* or aleatoric or epistemic or dropout or Bayes* or "conformal prediction" or "variational inference" or "temperature scaling" or platt or entropy):ti,ab or (monte NEXT carlo*):ti,ab | 28181 |
| #6 | {or #4-#5} | 28249 |
| #7 | #3 and #6 | 609 |
| #8 | MeSH descriptor: [Radiotherapy] explode all trees | 10381 |
| #9 | MeSH descriptor: [Radiotherapy Planning, Computer-Assisted] explode all trees | 467 |
| #10 | MeSH descriptor: [Radiation Oncology] explode all trees | 82 |
| #11 | (radiotherap* or irradiat* or radiat* or chemoradi* or radiochemo* or "intensity modulated" or IMRT or EBRT or photon* or proton* or radiosurgery or "radio-surgery" or brachytherapy or "brachy-therapy"):ti,ab or (((radio NEXT therap*) or (radio NEXT chemo*) or (chemo NEXT radi*))):ti,ab | 63161 |
| #12 | {or #8-#11} | 64059 |
| #13 | #7 and #12 | 42 |

###

### Web of Science Core Collection (Clarivate)

Entitlements: WOS.IC: 1993 to 2023; WOS.CCR: 1985 to 2023; WOS.SCI: 1900 to 2023; WOS.AHCI: 1975 to 2023; WOS.BHCI: 2005 to 2023; WOS.BSCI: 2005 to 2023; WOS.ESCI: 2005 to 2023; WOS.ISTP: 1990 to 2023; WOS.SSCI: 1900 to 2023; WOS.ISSHP: 1990 to 2023

| ID | Search | Results |
| --- | --- | --- |
| #1 | TI=((artificial or machine or deep) NEAR/1 (learning or intelligence)) OR AB=((artificial or machine or deep) NEAR/1 (learning or intelligence)) | 560769 |
| #2 | TI=("neural net*" OR "neural net*" or "support vector" or "decision tree" or "random forest" or "gradient boost*" or bagging or ensemble or radiom*) OR AB=("neural net*" or "support vector" or "decision tree" or "random forest" or "gradient boost*" or bagging or ensemble or radiom*) | 988580 |
| #3 | #2 OR #1 | 1344163 |
| #4 | TI=(uncertain* or aleatoric or epistemic or "monte carlo*" or dropout or Bayes* or "conformal prediction" or "variational inference" or "temperature scaling" or platt or entropy) OR AB=(uncertain* or aleatoric or epistemic or "monte carlo*" or dropout or Bayes* or "conformal prediction" or "variational inference" or "temperature scaling" or platt or entropy) | 1533339 |
| #5 | TI=(radiotherap* or "radio-therap*" or irradiat* or radiat* or chemoradi* or radiochemo* or "chemo-radi*" or "radio-chemo*" or "intensity modulated" or IMRT or EBRT or photon* or proton* or radiosurgery or "radio-surgery" or brachytherapy or "brachy-therapy") OR AB=(radiotherap* or "radio-therap*" or irradiat* or radiat* or chemoradi* or radiochemo* or "chemo-radi*" or "radio-chemo*" or "intensity modulated" or IMRT or EBRT or photon* or proton* or radiosurgery or "radio-surgery" or brachytherapy or "brachy-therapy") | 2578649 |
| #6 | #3 AND #4 AND #5 | 4358 |

###

### Web of Science Preprint Citation Index (Clarivate)

| ID | Search | Results |
| --- | --- | --- |
| #1 | TI=((artificial or machine or deep) NEAR/1 (learning or intelligence)) OR AB=((artificial or machine or deep) NEAR/1 (learning or intelligence)) | 79524 |
| #2 | TI=("neural net*" OR "neural net*" or "support vector" or "decision tree" or "random forest" or "gradient boost*" or bagging or ensemble or radiom*) OR AB=("neural net*" or "support vector" or "decision tree" or "random forest" or "gradient boost*" or bagging or ensemble or radiom*) | 99437 |
| #3 | #2 OR #1 | 153713 |
| #4 | TI=(uncertain* or aleatoric or epistemic or "monte carlo*" or dropout or Bayes* or "conformal prediction" or "variational inference" or "temperature scaling" or platt or entropy) OR AB=(uncertain* or aleatoric or epistemic or "monte carlo*" or dropout or Bayes* or "conformal prediction" or "variational inference" or "temperature scaling" or platt or entropy) | 147982 |
| #5 | TI=(radiotherap* or "radio-therap*" or irradiat* or radiat* or chemoradi* or radiochemo* or "chemo-radi*" or "radio-chemo*" or "intensity modulated" or IMRT or EBRT or photon* or proton* or radiosurgery or "radio-surgery" or brachytherapy or "brachy-therapy") OR AB=(radiotherap* or "radio-therap*" or irradiat* or radiat* or chemoradi* or radiochemo* or "chemo-radi*" or "radio-chemo*" or "intensity modulated" or IMRT or EBRT or photon* or proton* or radiosurgery or "radio-surgery" or brachytherapy or "brachy-therapy") | 142649 |
| #6 | #3 AND #4 AND #5 | 428 |

###

### Google Scholar (first 200 results)

(artificial learning OR machine learning OR deep learning OR artificial intelligence OR machine intelligence OR deep intelligence OR neural network OR neural networks OR neural networking OR support vector OR support vectors OR decision tree OR decision trees OR random forest OR gradient boost OR gradient boosts OR bagging OR ensemble OR radiomic OR radiometric OR radiomorhometric) AND (uncertainty OR aleatoric OR epistemic OR "monte carlo*" OR dropout OR Bayes OR "conformal prediction" OR "variational inference" OR "temperature scaling" OR platt OR entropy) AND (radiotherapy OR irradiation OR radiation OR chemoradiation OR radiochemotherapy OR "intensity modulated" OR IMRT OR EBRT OR photon* OR proton* OR radiosurgery OR brachytherapy)

### Key Articles

To ensure a comprehensive inclusion of relevant articles, the following PubMed IDs were used as “key articles” in shaping our initial search queries: “33179605" or "33503599" or "33778184" or "34111573" or "36112996" or "36484346" or "36865296" or "37414257" or "37820691".

## Appendix D: Additional data extraction for contouring studies investigating failure detection

The assessment of methodology correctness or success for uncertainty quantification in radiotherapy is highly subjective. Moreover, given the diverse nature of radiotherapy applications covered, there is unfortunately no one-size-fits-all approach for evaluating uncertainty quantification methodology for the papers in our review. However, to further analyze the suitability of current methodology for contouring studies with failure detection applications (the most prevalent application in our review), we utilized reference data from the ValUES framework by Kahl et al. (doi: 10.48550/arXiv.2401.0850). Based on their findings, we focused on two key aspects:

1. Uncertainty quantification metrics: Predictive uncertainty metrics (e.g., total predictive entropy) consistently perform above average for failure detection applications. This aligns with underlying mathematical theory. Meanwhile, aleatoric (e.g., expected entropy) and epistemic (e.g., mutual information) uncertainty metrics excel in different scenarios (dependent on dataset properties). Given these findings, our goal is to determine whether studies included at least one metric of predictive uncertainty in their analysis.
2. Uncertainty quantification methods: Ensembles most consistently outperform other uncertainty quantification methods for failure detection. Given this finding, our goal is to determine whether studies employed an ensemble technique in their analysis.

We did not include the choice of aggregation method in our analysis, as Kahl et al. reported mixed results for failure detection in this area.

Using these criteria, we aimed to determine how many of the 20 contouring studies in our review that investigate failure detection applications utilized the preferred metrics and methods as recommended by the ValUES framework.

**Table D1** presents the 20 contouring studies with relevant extracted data. Of these, 12 studies (60%) employed predictive uncertainty metrics, 5 studies (25%) implemented ensemble methods, and 4 studies (20%) combined both approaches by using predictive uncertainty metrics and implementing ensembles.

**Table D1.** Contouring studies with failure detection (FD) applications categorized by preferred ValUES uncertainty quantification metric (i.e., predictive uncertainty) and uncertainty quantification method (i.e., ensemble).

| **Study ID** | **Title** | **ValUES FD predictive uncertainty metric** | **ValUES FD ensemble model** |
| --- | --- | --- | --- |
| Jungo 2018a | Uncertainty-driven Sanity Check: Application to Postoperative Brain Tumor Cavity Segmentation | Yes | No |
| Jungo 2018b | On the Effect of Inter-observer Variability for a Reliable Estimation of Uncertainty of Medical Image Segmentation | Yes | No |
| Ninomiya 2018 | Bayesian delineation framework of clinical target volumes for prostate cancer radiotherapy using an anatomical-features-based machine learning technique | No | No |
| Qin 2018 | Superpixel-based and boundary-sensitive convolutional neural network for automated liver segmentation. | Yes | No |
| Chen 2020 | CNN-Based Quality Assurance for Automatic Segmentation of Breast Cancer in Radiotherapy. | No | No |
| Gustafsson 2020 | Development and evaluation of a deep learning based artificial intelligence for automatic identification of gold fiducial markers in an MRI-only prostate radiotherapy workflow. | No | No |
| Hansch 2020 | Hippocampus segmentation in CT using deep learning: impact of MR versus CT-based training contours. | Yes | No |
| Balagopal 2021 | A deep learning-based framework for segmenting invisible clinical target volumes with estimated uncertainties for post-operative prostate cancer radiotherapy. | No | No |
| Lei 2021 | Automatic segmentation of organs-at-risk from head-and-neck CT using separable convolutional neural network with hard-region-weighted loss | Yes | Yes |
| Mei 2021 | Automatic segmentation of gross target volume of nasopharynx cancer using ensemble of multiscale deep neural networks with spatial attention | Yes | Yes |
| vanRooij 2021 | Using Spatial Probability Maps to Highlight Potential Inaccuracies in Deep Learning-Based Contours: Facilitating Online Adaptive Radiation Therapy. | No | No |
| Mody 2022a | Comparing Bayesian Models for Organ Contouring in Head and Neck Radiotherapy | Yes | No |
| Mody 2022b | Improving Error Detection in Deep Learning Based Radiotherapy Autocontouring Using Bayesian Uncertainty | Yes | No |
| Zabihollahy 2022 | Fully automated segmentation of clinical target volume in cervical cancer from magnetic resonance imaging with convolutional neural network. | No | No |
| Cubero 2023 | EXPLORING UNCERTAINTY FOR CLINICAL ACCEPTABILITY IN HEAD AND NECK DEEP LEARNING-BASED OAR SEGMENTATION | Yes | No |
| DeBiase 2023 | Deep learning aided oropharyngeal cancer segmentation with adaptive thresholding for predicted tumor probability in FDG PET and CT images. | No | Yes |
| Ebadi 2023 | CBCT-guided adaptive radiotherapy using self-supervised sequential domain adaptation with uncertainty estimation. | Yes | Yes |
| Min 2023 | Clinical target volume delineation quality assurance for MRI-guided prostate radiotherapy using deep learning with uncertainty estimation. | No | No |
| Outeiral 2023 | A network score-based metric to optimize the quality assurance of automatic radiotherapy target segmentations | Yes | No |
| Sahlsten 2023 | Application of simultaneous uncertainty quantification for image segmentation with probabilistic deep learning: Performance benchmarking of oropharyngeal cancer target delineation as a use-case. | Yes | Yes |
